# Etude du pronostic maternel et périnatal au cours de l’accouchement chez l’adolescente à Lubumbashi, République Démocratique du Congo

**DOI:** 10.11604/pamj.2017.26.182.9479

**Published:** 2017-03-29

**Authors:** Prosper Kakudji Luhete, Olivier Mukuku, Albert Mwembo Tambwe, Prosper Kalenga Muenze Kayamba

**Affiliations:** 1 Département de Gynécologie-Obstétrique, Faculté de Médecine, Université de Lubumbashi, RD Congo

**Keywords:** Adolescence, accouchement, pronostic maternel et périnatal, Lubumbashi, Adolescent girls, vaginal delivery, maternal and perinatal prognosis, Lubumbashi

## Abstract

**Introduction:**

L’objectif de cette étude était de déterminer la fréquence et d’évaluer le pronostic maternel et périnatal lors de l’accouchement chez les adolescentes dans la ville de Lubumbashi.

**Méthodes:**

C’était une étude cas-témoin des accouchées d’une grossesse monofoetale de Décembre 2013 à Mai 2014 dans 10 maternités de référence à Lubumbashi (RD Congo). Les adolescentes (< 20 ans) ont été comparées aux femmes âgées de 20-34 ans. Les paramètres sociodémographiques maternels, la morbi-mortalité maternelle et périnatale ont été analysées. Les statistiques usuelles et la régression logistique ont été utilisées pour analyser les résultats. Le seuil de signification a été fixé à une valeur de p<0,05.

**Résultats:**

**L**a fréquence d’accouchement chez les adolescentes était de 7,7%. Nous avons observé que la césarienne (ORa=1,9 (1,1-3,1)), l’épisiotomie (ORa=4,2 (2,9-5,9)), la délivrance pathologique (ORa= 2,7 (1,1-6,5)), l’éclampsie (ORa= 4,4 (1,3-14,5)) et le faible poids de naissance (ORa=2,0 (1,3-3,0)) ont été significativement plus élevés chez les adolescentes que chez les adultes.

**Conclusion:**

**L**’accouchement chez les adolescentes, comparativement à celui de femmes âgées de 20-34 ans, reste associé à un mauvais pronostic. D’où l’organisation des séances de sensibilisation pour une meilleure fréquentation des services consultations prénatales, une optimisation du dépistage, de la surveillance et de la prévention des pathologies de la grossesse chez les adolescentes s’avère importante et urgente.

**Introduction:**

This study aimed to determine the frequency and to assess maternal and perinatal prognosis for vaginal delivery in adolescent girls in the city of Lubumbashi.

**Methods:**

We conducted a case-control study of vaginal deliveries in singleton pregnancy in 10 referral hospitals in Lubumbashi (DR Congo) from December 2013 to May 2014. Adolescent girls (< 20 years) were compared to older women aged 20-34 years. Maternal sociodemographic parameters, morbi-maternal and perinatal mortality were analyzed. Usual statistics and logistic regression were used to analyze the results. The significance level was set at p <0.05.

**Results:**

Vaginal delivery rate among adolescent girls was 7.7%. Cesarean section (OR=1.9 (1.1-3.1)), episiotomy (OR=4.2 (2.9-5.9)), pathological delivery (OR=2.7 (1.1-6.5)), eclampsia (OR=4.4 (1.3-14.5)) and low birth weight (OR=2.0 (1.3-3.0)) were significantly higher among adolescent girls than in adults.

**Conclusion:**

Vaginal delivery in adolescent girls, compared to that of older women aged 20-34 years, is associated with a poor prognosis. Hence the importance and the urgent need to implement awareness sessions to increase attendance to prenatal consultation services, for screening optimization, monitoring and prevention for pregnancy pathologies in adolescent girls.

## Introduction

Selon l’Organisation Mondiale de la Santé (OMS), un adolescent est tout celui dont l’âge chronologique se situe entre 10 à 19 ans [1]. En 2012, l’OMS [2] a estimé que près de 16 millions de jeunes filles âgées de 15 à 19 ans et 2 millions de jeunes filles de moins de 15 ans accouchent chaque année dans le monde et la moitié de toutes les naissances chez des adolescentes survient dans sept pays seulement: le Bangladesh, le Brésil, les États-Unis d’Amérique, l’Éthiopie, l’Inde, le Nigéria et la République démocratique du Congo (RDC). A l’instar d'autres pays en développement, la RDC enregistre un taux élevé de grossesses chez les adolescentes. Selon l'Enquête Démographique et de Santé (EDS), près d’un quart des adolescentes congolaises (24%) ont déjà commencé leur vie féconde (près de 19% ont déjà eu au moins un enfant et 5% sont enceintes d’un premier enfant) et les proportions d’adolescentes ayant commencé leur vie féconde augmentent rapidement avec l’âge, passant de 6% à 15 ans à 47% à 19 ans, âge auquel 42% des jeunes filles ont déjà eu au moins un enfant [3]. La grossesse et l’accouchement chez l’adolescente portent un très haut risque de morbidité et mortalité. Ceci serait lié aux caractéristiques physiologiques et sociologiques des adolescentes. Elles totalisent 23% de la charge globale de morbidité (en années de vie ajustées sur l’incapacité) du fait de la grossesse et de l’accouchement [2]. Les grossesses chez l'adolescente présentent des risques accrus pour la santé de la mère tels que l'anémie, l'hypertension, l'éclampsie et les troubles dépressifs [4-6] mais aussi pour celle de l'enfant dont un risque accru pour celui-ci d'avoir un faible poids à la naissance, d'être prématuré, de naître déprimé et, par conséquent, d'être exposé à une morbi-mortalité plus importante pendant l'enfance [4, 7]. Dans les pays à revenu faible ou moyen, les complications de la grossesse et de l’accouchement sont l’une des principales causes de décès pour les jeunes filles âgées de 15 à 19 ans. En plus, les morts naissances et les décès néonatals sont 50% plus nombreux chez les enfants de mères adolescentes que parmi ceux de femmes âgées de 20 à 29 ans [2, 8]. Si l’ampleur de ce problème est largement appréhendée dans la plupart des pays voire des régions du monde, ceci n’est pourtant pas le cas pour la ville de Lubumbashi, en RDC. Suite à des résultats contradictoires des études épidémiologiques précédentes menées ailleurs [9-12] et un manque de données récentes en ce qui concerne la ville de Lubumbashi, nous avons mené cette étude multicentrique avait pour objectifs de déterminer la fréquence de l’accouchement chez les adolescentes, de décrire leurs caractéristiques socio-démographiques et d’évaluer le pronostic maternel et périnatal lors de l’accouchement chez les adolescentes dans la ville de Lubumbashi.

## Méthodes

**Figure 1:**
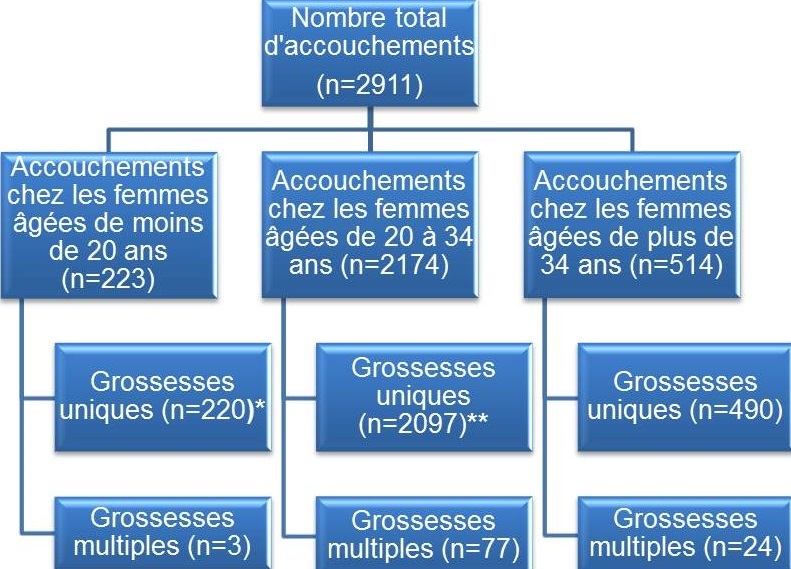
Distribution des accouchées enrôlées dans l’étude

Il s’agit d’une étude cas-témoin menée sur la période allant du 1er décembre 2013 au 31 mai 2014. Au cours de cette période d’étude, nous avons enregistré tous les accouchements réalisés dans les maternités des 10 hôpitaux généraux de référence (HGR) de la ville de Lubumbashi en RDC (hôpital militaire de Ruashi, Cliniques Universitaires, hôpital Jason Sendwe, HGR Katuba, HGR Kenya, HGR Kamalondo, HGR Kisanga, HGR Kampemba, hôpital Gécamines-Sud et hôpital SNCC). Ces hôpitaux sont répartis dans les 7 communes que compte la ville de Lubumbashi. Toutes les femmes qui se sont présentées dans ces formations sanitaires choisies pour un accouchement ont été incluses dans l'étude quel que soit le lieu de suivi de la grossesse. Au total, 2911 accouchements ont été enregistré exhaustivement dont 2317 ont concerné des femmes âgées de moins de 35 ans avec grossesses monofoetales ayant constitué notre échantillon (Figure 1). Ces femmes ont été réparties en deux groupes, en fonction de leur âge : un groupe comprenant les femmes âgées de moins de 20 ans et un second groupe réunissant les femmes âgées de 20 à 34 ans. Les caractéristiques sociodémographiques maternelles, les paramètres en rapport avec la morbi-mortalité maternelle et périnatale ont été recueillis par le personnel effectuant habituellement l'accouchement dans les sites d'enquête. Un entretien a permis de recueillir les caractéristiques sociodémographiques de la patiente ainsi que les antécédents obstétricaux. Une fiche d’enquête individuelle avait été élaborée à cet effet et la recherche de données complémentaires a été réalisée dans le dossier obstétrical.

*Caractéristiques sociodémographiques maternelles:* l’âge maternel (femmes de moins de 20 ans (maternité précoce) et de 20 à 34 ans (maternité normale)), la parité, le statut matrimonial (mariées, non mariées, divorcées et veuves), la profession (élève/étudiante, ménagère, employée, activité rémunératrice autonome), le niveau d’étude (non scolarisée, primaire, secondaire et supérieur) et le suivi de la grossesse (une grossesse était considérée comme non suivie si aucune consultation prénatale (CPN) n’avait eu lieu, mal suivie si le nombre de CPN était inférieur à 4 et bien suivie si ce nombre était supérieur ou égal à 4).

*Paramètres en rapport avec la morbi-mortalité maternelle:* l’accouchement par césarienne, la présentation foetale vicieuse (non céphalique de sommet), l’épisiotomie, l’anémie post-partale, la notion de transfusion, l’éclampsie, la délivrance pathologique, les lésions de parties molles, les complications maternelles, le décès maternel. La délivrance était considérée pathologique lorsque l’on notait la rétention placentaire et/ou l’hémorragie de la délivrance. Les lésions des parties molles réunissaient les déchirures cervicale, vaginale et périnéale. L'anémie a été établie sur base des signes cliniques et/ou sur base d'un taux d'hémoglobine inférieur à 11 g/l quand cet examen était disponible. L’éclampsie était définie comme un accident aigu paroxystique compliquant la toxémie gravidique, caractérisé par des accès convulsifs à répétition d’apparition brutale ou succédant à une phase prémonitoire qui associe signes neurologiques et digestifs et pouvant survenir pendant la grossesse (le plus souvent au cours du 3^ème^ trimestre), parfois pendant l’accouchement ou dans les 48 heures après la délivrance. Dans le paramètre « *complications maternelles* », nous avons regroupé toute complication maternelle notée en post-partum dont les infections, l’anémie, l’éclampsie, l’hémorragie, la psychose, les thrombo-phlébites, …

*Paramètres en rapport avec la morbi-mortalité périnatale:* le faible poids de naissance (< 2500 grammes), la dépression néonatale (score d’Apgar à la cinquième minute < 7), la prématurité, la mort foetale in utéro, le décès périnatal. L’âge maternel est considéré ici comme variable dépendante et les paramètres en rapport avec la morbi-mortalité maternelle et périnatale constituent les variables indépendantes. Les caractéristiques sociodémographiques et la morbi-mortalité maternelle et périnatale des accouchées de moins de 20 ans ont été comparées à celles des accouchées âgées de 20 à 34 ans. Le test de khi2 d’indépendance (corrigé si nécessaire) ou du test exact de Fisher pour les variables qualitatives et le test de Student pour les variables quantitatives. L’odds ratio (OR) et ses intervalles de confiance à 95% (IC95%) ont été calculés. Le seuil de significativité était fixé à p < 0,05. L’ajustement a été fait à l’aide de la régression logistique. Les variable atteignant un degré de significativité de p < 0,2 ont été retenus comme variables candidates et ont été introduits dans une série de modèles de régression logistique par la méthode d’entrée en bloc. Les analyses ont été réalisées à l'aide des logiciels Epi Info 7.1 et Stata 12. La recherche pour réaliser ce travail a été autorisée par le comité d’éthique de l’Université de Lubumbashi. Un consentement libre et éclairé de toutes les personnes impliquées dans cette étude a été obtenu verbalement.

## Résultats

**Fréquence**: sur un total de 2911 accouchées consécutivement enregistrées au cours de la période d'étude, nous avons répertorié 223 accouchées âgées de moins de 20 ans, soit une fréquence de 7,7% (Figure 1).

### Caractéristiques socio-démographiques, suivi de la grossesse et notion de référence de la population étudiée

Le Tableau 1 présente les caractéristiques socio-démographiques, le suivi de la grossesse et la notion de référence de la population de l'étude. L’âge moyen était de 17,6±1,2 ans chez les accouchées adolescentes et de 27,0±4,1 ans chez les accouchées adultes. La moyenne de la parité était de 1,3±0,5 chez les accouchées adolescentes contre 3,4±1,9 chez celles âgées de 20 ans ou plus avec une différence statistiquement significative entre les deux groupes sur la comparaison de ces parités moyennes (p< 0,001). Dans la série, nous avons observé, dans le groupe des accouchées adolescentes, que 18,6% étaient célibataires, 7,7% étaient non scolarisées et près de 1,8% avaient un emploi contre respectivement 1,6%, 6,5% et 9,6% dans le groupe des accouchées adultes (p< 0,001). La proportion des accouchées adolescentes qui ont été référés est supérieure à celle des accouchées adultes (7,7 contre 5,1%) mais nous n’avons observé aucune différence statistiquement significative (p=0,1275). Quant au suivi de la grossesse, la moyenne de nombre de CPN était de 2,0±1,8 avec une proportion de 31,8% des grossesses non suivies chez les accouchées adolescentes alors qu’elle était de 2,6±1,9 avec une proportion de grossesses non suivies de 20,5% chez les accouchées adultes. Nous avons observé une différence statistiquement significative lorsque nous comparons les deux moyennes ainsi que les deux taux (p<0,001).

**Tableau 1:** caractéristiques sociodémographiques, le suivi de la grossesse et la notion de référence chez les adolescentes à Lubumbashi

Paramètres	<20 ans (n=220)		20-34 ans (n=2097)		Total (n=2317)		p
	n	(%)	n	(%)	n	(%)	
**Age maternel (ans)**							
Moyenne	17,6±1,2		27,0±4,1		26,2±4,8		-
**Parité**							
Moyenne	1,3±0,5		3,4±1,9		3,2±1,9		<0,001
(Extrêmes)	(1 – 4)		(1 – 12)		(1 – 12)		
1	174	(79,1)	384	(18,3)	558	(24,1)	<0,001
2-4	46	(20,9)	1156	(55,1)	1202	(51,9)	
≥5	0	(0,0)	557	(26,6)	557	(24,0)	
**Etat-civil**							
Célibataire	41	(18,6)	33	(1,6)	74	(3,2)	<0,001
Mariée	179	(81,4)	2064	(98,4)	2243	(96,8)	
**Niveau d’études**							
Non scolarisée	17	(7,7)	136	(6,5)	153	(6,6)	<0,001
Primaire	13	(5,9)	39	(1,9)	52	(2,2)	
Secondaire	174	(79,1)	1047	(49,9)	1221	(52,7)	
Supérieur	16	(7,3)	875	(41,7)	891	(38,5)	
**Profession**							
Travailleuse	4	(1,8)	202	(9,6)	206	(8,9)	<0,001
Etudiante	24	(10,9)	45	(2,1)	69	(3,0)	
Sans emploi	192	(87,3)	1850	(88,3)	2042	(88,1)	
**Référence**							
Référée	17	(7,7)	106	(5,1)	123	(5,3)	0,128
Non référée	203	(92,3)	1991	(94,9)	2194	(94,7)	
**Nombre de CPN**							
Moyenne	2,0±1,8		2,6±1,9		2,6±1,9		<0,001
(Extrêmes)	(0 – 7)		(0 – 14)		(0 – 14)		
0	70	(31,8)	430	(20,5)	500	(21,6)	<0,001
1-3	107	(48,6)	1058	(50,5)	1165	(50,3)	
≥4	43	(19,5)	609	(29,0)	652	(28,1)	

CPN : consultations prénatales

### Morbidité et mortalité maternelles

Les paramètres en rapport avec la morbidité ainsi que la mortalité maternelles sont présentés dans le Tableau 2. Chez 5,5% des mères adolescentes, la présentation foetale était vicieuse contre 3,4% chez les mères adultes; la différence observée n’était pas statistiquement significative. La césarienne était le mode d’accouchement chez 11,4% d’adolescentes contre chez 7,4% d’adultes (OR ajusté=1,9 (1,1-3,1)). Les déchirures des parties molles ont été observées dans 10,0% des cas chez les adolescents contre 7,6% chez les non-adolescents (p=0,2329). Chez les accouchées de moins de 20 ans, la proportion d’épisiotomie était de 29,1% contre 10,2% chez celles âgées de 20 ans ou plus. Nous avons observé qu’elles présentent un rapport de côtes élevé de subir une épisiotomie comparativement à ces dernières (OR ajusté=4,2 [2, 9-5, 9]). Les mères adolescentes présentent plus de risque d’avoir une délivrance pathologique lors de l’accouchement que les mères adultes (4,1% contre 1,3%; OR ajusté= 2,7 (1,1-6,5)). Concernant la présence de complications maternelles en période post-partale, nous avons noté moins de complications chez les mères adultes que chez les mères adolescentes (respectivement 5,5 et 2,0%) et ces dernières présentent un rapport de côtes de près de 3 fois plus élevé par rapport à leurs homologues (OR=2,8 (1,5-5,4)). L’éclampsie notée à l’accouchement ou en post-partum était plus enregistré chez les adolescentes que chez les adultes avec des proportions respectives de 3,6 et 0,6%. Comparée à une mère adulte, une mère adolescente présente plus de chance de faire l’éclampsie en péripartum (OR ajusté=4,4 (1,3-14,5)). Par ailleurs, la survenue de l’anémie post-partale n’a pas montré de différence statistiquement significative entre ces deux groupes (p=0,0813). En recherchant la notion de transfusion lors de l’accouchement, nous avons noté les accouchées adolescentes étaient les plus transfusées que les accouchées adultes (1,4% contre 0,7%) mais aucune différence statistique n’a été notée en comparant ces deux proportions (p=0,2144). Enfin, 7 décès maternels ont été notés dans la population d’étude dont 1 soit 0,5% dans le groupe d’accouchées adolescentes et 6 soit 0,3% dans le groupe d’accouchées adultes ; la différence entre ces deux proportions n’était pas statistiquement significative (p=0,5030).

**Tableau 2:** pronostic maternel et périnatal chez les adolescentes

Paramètre	<20 ans (n=220)		20-34 ans (n=2097)		OR brut [IC 95%]	OR ajusté [IC 95%]
	n	(%)	n	(%)		
Présentation vicieuse	12	(5,5)	72	(3,4)	1,6 [0,8-3,0]	-
Césarienne	25	(11,4)	156	(7,4)	1,6 [1,0-2,5]	1,9 [1,1-3,1]
Lésions de parties molles	22	(10,0)	159	(7,6)	1,4 [0,8-2,2]	-
Episiotomie	64	(29,1)	213	(10,2)	3,6 [2,6-5,0]	4,2 [2,9-5,9]
Délivrance pathologique	9	(4,1)	27	(1,3)	3,2 [1,3-7,3]	2,7 [1,1-6,5]
Présence de complications maternelles	12	(5,5)	42	(2,0)	2,8 [1,5-5,4]	-
Eclampsie	8	(3,6)	12	(0,6)	6,6 [2,6-16,2]	4,4 [1,3-14,5]
Anémie post-partale	5	(2,3)	20	(1,0)	2,4 [0,7-6,7]	-
Transfusion	3	(1,4)	14	(0,7)	2,1 [0,4-7,4]	-
Décès maternel	1	(0,5)	6	(0,3)	1,6 [0,0-13,2]	-
Score d’Apgar à la 5è minute <7	18	(8,2)	117	(5,6)	1,5 [0,9-2,5]	-
Naissance prématurée (<37 SA)*	25	(12,7)	138	(6,9)	1,9 [1,3-3,0]	-
PN <2500g	42	(19,6)	222	(10,7)	2,0 [1,4-2,9]	2,0 [1,3-3,0]
Décès périnatal	14	(6,4)	68	(3,2)	2,0 [1,1-3,7]	-

g : grammes ; * l’âge gestationnel était connu chez 197 adolescentes et chez 2002 adultes.

### Morbidité et mortalité périnatales

Les paramètres en rapport avec la morbidité ainsi que la mortalité périnatales sont présentés dans le Tableau 2. Les résultats des analyses dans cette série montrent que dans les 2 groupes étudiés, la proportion des FPN est statistiquement plus élevée dans le groupe des mères adolescentes que dans celui des mères âgées de 20 ans ou plus ; la chance que présentent ces mères adolescentes d’accoucher un FPN est de 2 fois (19,6 contre 10,7% ; OR ajusté=2,0 (1,3-3,0)). La moyenne de PN est de 3019±556 grammes chez les nouveau-nés des adolescentes alors qu’elle est de 3175±520 grammes chez ceux nés des adultes. Nous avons observé une différence statistique significative entre ces deux moyennes (p< 0,001). Douze virgule sept pourcent des enfants nés des mères adolescentes avaient un enfant prématuré contre 6,9% de ceux nés des mères adultes (p=0,0025) signifiant un risque de près de deux fois pour un enfant né d’une mère adolescente de naitre prématurément (OR=1,9 (1,3-3,0)). La proportion de nouveau-nés déprimés à la 5^ème^ minute de vie (score d'Apgar < 7) était de 8,2% dans le groupe des adolescentes contre 5,6% dans le groupe des adultes. En comparant ces deux proportions, la différence n’est pas statistiquement significative (p=0,1566). Les moyennes du score d'Apgar à la 5^ème^ minute étaient de 9,0±1,3 et 9,2±1,1 respectivement chez les nouveau-nés des mères adolescentes et chez ceux nés des mères adultes; la comparaison entre ces deux moyennes n’est pas statistiquement significative (p=0,0869). S’agissant du décès périnatal, nous avons enregistré 6,4% de décès chez les enfants des mères adolescentes contre 3,2% chez ceux des mères adultes avec une différence statistiquement significative (p=0,0284) signifiant que la chance de décès périnatal est de deux fois élevée en défaveur des enfants nés des mères âgées de moins de 20 ans (OR=2,0 (1,1-3,7)).

## Discussion

### Fréquence

Cette étude multicentrique rapporte une fréquence de 7,7%. En 1999, dans une étude monocentrique menée aux Cliniques Universitaires de Lubumbashi (RDC), Tambwe trouve 13,9% [13]. Notre fréquence est proche à celles trouvées dans d’autres études menées dans des zones urbaines en Afrique qui varient autour de 7-13% [14-16]. En milieu rural, en 2006, Tebeu obtient 26,5% dans le Nord du Cameroun [17]. Plusieurs facteurs peuvent expliquer la différence entre les différents milieux en ce qui concerne le taux d’accouchement chez les adolescentes : la pauvreté, l’analphabétisme, le mariage précoce lié aux facteurs cultures, ethniques et religieux [18]. C’est ainsi l'éducation de la fille est promu dans le milieu urbain contrairement au milieu rural où celle-ci n’est pas jugée prioritaire et les mariages précoces sont encouragés. Mais il faut souligner que des fréquences très faibles (inférieur à 2%) ont été notées à Enugu (Nigeria) et à Kuala Lumpur (Malaisie) [9, 19]. Les auteurs notent que la faible incidence peut être attribuée à une nouvelle tendance dans la zone où les adolescentes enceintes non mariées sont encouragés par certaines organisations non gouvernementales pour accoucher souvent dans des établissements médicaux privés et les donner pour adoption comme une alternative à l'avortement dans les grossesses non désirées; et de ce fait, beaucoup de ces grossesses ne sont pas signalés.

### Caractéristiques socio-démographiques et surveillance prénatale

Les accouchées adolescentes avaient un âge moyen de 17,6 ans et une moyenne de la parité de 1,3. Ces moyennes sont superposables à celles trouvées par d’autres auteurs [14, 20, 21]. Dans la série, les accouchées adolescentes étaient célibataires dans 18,6%, étaient non scolarisées 7,7% et seulement 1,8% avaient un emploi. Ces proportions sont statistiquement différentes à celles des accouchées adultes et plaident plus en défaveur des adolescentes. Plusieurs auteurs ont fait un constat identique en ce qui concerne les aspects socio-démographiques défavorables et le mauvais ou manque de suivi prénatal des adolescentes [22-25]. Le suivi prénatal est la période privilégiée où les grossesses à risque sont décelées en vue d’une prise en charge. Nos résultats montrent une différence significative lorsque nous comparons les moyennes de nombre de CPN (2,0 versus 2,6) ainsi que les taux de grossesses non suivies (31,8% versus 20,5%) chez les accouchées adolescentes et chez les accouchées adultes. Dans l’ensemble, 80,5% des accouchées adolescentes n’ont pas bénéficié de CPN bien suivies, ce qui pourrait avoir contribué aux complications de la grossesse. Tambwe, rapporte un taux de grossesses non suivies chez les adolescentes de 30,2% [13]. Selon l’OMS, dans les pays en développement, les jeunes femmes enceintes se présentent souvent tardivement aux CPN (dans le deuxième ou troisième trimestre de grossesse) ou ne se présentent même pas pour des soins prénatals. Les raisons évoquées pour cette apathie vers les services de soins prénatals comprennent l'ignorance de l'importance des soins prénatals (surtout chez les non inscrites), le manque de soutien familial ou social, la non disponibilité des services de soins prénatals, la pauvreté, des remarques désagréables des agents de santé vers les adolescentes non mariées qui sont enceintes, et tentent de se soustraire du regard du public puisque certaines cliniques manquent d’intimité [26], mais aussi la crainte de dépistage du VIH [27]. L'association entre l’accouchement chez l'adolescence et le pronostic maternel et périnatal défavorable pourrait être expliquée en parti par l'environnement social délétère [28].

### Morbidité et mortalité maternelles

L’étude montre que la présentation fœtale vicieuse (non céphalique de sommet), les lésions des parties molles, l’anémie post-partale et la notion de transfusion lors de l’accouchement n’ont pas été statistiquement significative liés au jeune âge (p>0,05). Cependant, comparativement à leurs homologues adultes, les accouchées adolescentes avaient statistiquement un risque élevé d’accoucher par césarienne, de subir une épisiotomie, d’avoir une délivrance pathologique lors de l’accouchement, de présenter l’éclampsie en péripartum et de présenter, de manière générale, de complications maternelles en période post-partale. L’étude relève que la césarienne a été significativement plus fréquente chez les moins de 20 ans que chez les âgées de 20-34 ans (ORa=1,9). Cette tendance a été retrouvée dans certaines études [29-31]. Dans une étude menée en Iran, Maryam constate que le taux élevé de césarienne était associé à l’âge inférieur à 17 ans [32]. Chez l’adolescente, le bassin croit plus lentement et progressivement jusqu’à l’âge avancé. De plus l’acquisition de la taille adulte n’implique pas une croissance équivalente du bassin car « le bassin ne termine définitivement sa configuration que vers la 25^ème^ année bien que les formes adultes sont atteintes vers l’âge de 16 ans » [33]. Cette immaturité du bassin est responsable des anomalies du bassin, bassin limite, bassin généralement rétréci chez l’adolescente. Et ce dernier est à son tour responsable des complications obstétricales plus fréquentes, principalement en dessous de 15 ans [34, 35]. Par contre, nos résultats contrastent avec ceux trouvés dans d’autres études: certaines enregistrent un taux significativement élevé des césariennes chez les adultes [35-39] et d’autres ne trouvent aucune différence significative entre les adolescentes et les adultes [40]. Le taux d’éclampsie est inversement proportionnel à l’âge de la femme dans notre série et la survenue de l'éclampsie a été plus fréquente chez les moins de 20 ans (ORa=4,4). Cette association entre l’éclampsie et l’adolescente a été aussi retrouvée dans d’autres études [37, 40, 41]. Pratiquement, plusieurs auteurs reconnaissent la fréquence élevée de l’hypertension artérielle chez la femme très jeune et mentionnent l’immaturité biologique et endocrinienne, la primigestité et le manque de suivi prénatal comme facteurs déterminants dans la survenue des syndromes vasculo-rénaux [4, 42-44], ce qui assombri ainsi le pronostic néonatal. L’épisiotomie était pratiquée 4 fois plus chez les adolescentes que chez les adultes (ORa=4,2). Ce constat est retrouvé dans plusieurs études [14, 19, 40] et l’immaturité du périnée pourrait bien expliquer ce taux élevé de l'épisiotomie notée [35]. En recherchant la notion de transfusion et celle de l’installation de l’anémie en postpartale, aucune différence significative entre les deux groupes n’a été notée. Nos résultats sont contradictoires avec ceux de Leppälahti [4] et de Mahavarkar [35] qui trouvent un risque élevé de développer une anémie au décours d’un accouchement dans le groupe des adolescentes. Mais ils sont comparables à ceux observés par Sulaiman [9], Iacobelli [21] et Fouelifack [14]. Ce dernier pense que les saignements sont tributaires d'autres causes telles que les compétences techniques du personnel dirigeant l’accouchement, la gestion active de la troisième phase du travail ainsi que les processus physiologiques plutôt que l'âge de la parturiente. S’agissant de la mortalité maternelle, le taux de décès maternel n’était pas statistiquement différent entre les deux groupes. Plusieurs auteurs retrouvent, comme dans notre série, un taux de décès maternel statistiquement indifférent entre les adolescentes et les adultes (p>0,05) [14, 29].

### Morbidité et mortalité périnatales

Les résultats des analyses dans cette série montrent, comparativement aux mères âgées de 20 ans ou plus, que les mères adolescentes présentent un risque de 2 fois d’accoucher un FPN et un risque de près de deux fois avoir un prématuré. La majorité des auteurs sont unanimes sur le fait que les adolescentes donnent souvent naissance à des prématurés et des nouveau-nés hypotrophes et de manière globale des nouveau-nés de FPN [21, 35, 40, 41, 45-48]. Chez l’adolescente, l’immaturité physique de l’utérus (encore hypoplasique) est souvent mise en cause dans la naissance d’un prématuré et voire celle d’un FPN [49]. Outre cette immaturité physique, certains auteurs incriminent le régime alimentaire des adolescentes qui est généralement plus problématique que celui des adultes, par sa pauvreté, et par ses habitudes diététiques. La grossesse chez les adolescentes engendre des besoins nutritionnels et alimentaires spécifiques, différents de ceux de la grossesse des femmes adultes, car le corps adolescent n'a pas encore atteint la maturité, et continue sa croissance ; d’où les besoins de la grossesse s’ajoutent à ceux de la croissance [45, 50-52]. Les études sur les grossesses des adolescentes confirment la compétition entre le corps de la mère adolescente et le corps du foetus, cette compétition intéresse les besoins alimentaires, les nutriments, les vitamines et minéraux. Cela explique que les adolescentes ont deux fois plus de risques que les femmes adultes d'avoir des bébés à faible poids à la naissance, et plus de risques que les femmes adultes d'accoucher de prématurés [45, 52, 53]. L’étude montre que bien qu’il a été noté cliniquement une proportion élevé des nouveau-nés déprimés à la 5ème minute de vie (score d'Apgar < 7) chez les adolescentes par rapport aux adultes (8,2% contre 5,6%), l’analyse ne donne pas de différence statistiquement significative. Ceci rejoint le constat d’Usta [54]. Concernant la mortalité périnatale, un risque de deux fois de décès périnatal est retrouvé chez les nouveau-nés des mères adolescentes, résultats superposables à ceux retrouvés au Cameroun par Fouelifack [14]. Les risques accrus observés de mortalité périnatale chez les nouveau-nés des mères adolescentes sont compatibles avec ceux des enquêtes précédentes [7, 8, 55-58]. Il y a, cependant, un certain nombre d'études qui n'ont pas trouvé une association entre le jeune âge maternel et la mortalité périnatale [29, 38, 59, 60]. Les résultats de l’étude d’Olausson trouvent que les risques de mortalité néonatale et post-néonatale augmentent constamment avec la diminution de l'âge maternel [61].

## Conclusion

L’accouchement chez les adolescentes, comparativement à celui de femmes âgées de 20-34 ans, reste associé à un mauvais pronostic. Ces complications sont évitables, d´abord en diminuant la fécondité des adolescentes par une contraception bien menée et surtout, en cas de grossesse, en améliorant la qualité des soins prénatals, per partum et postnatals. L’organisation des séances de sensibilisation pour une meilleure fréquentation des services consultations prénatales, une optimisation du dépistage, de la surveillance et de la prévention des pathologies de la grossesse chez les adolescentes s’avère importante et urgente.

### Etat des connaissances actuelle sur le sujet

- L’accouchement chez l’adolescente constitue un problème majeur de santé publique en République Démocratique du Congo;
- La grossesse et l’accouchement chez l’adolescente portent un très haut risque de morbidité et mortalité lié aux caractéristiques physiologiques et sociologiques des adolescentes.

### Contribution de notre étude à la connaissance

- Aucune étude sur ce sujet n’a déjà été publiée antérieurement sur les facteurs de risque et le pronostic maternel et périnatal de l’accouchement chez l’adolescente dans notre contexte, à Lubumbashi, République Démocratique du Congo;
- L'étude proposée est la première étude globale et multicentrique dans notre pays, intégrant une analyse multivariée permettant d’évaluer le pronostic maternel et périnatal dans notre contexte.
